# Endotracheal intubation under video laryngoscopic guidance during upper gastrointestinal endoscopic surgery in the left lateral position: A randomized controlled trial

**DOI:** 10.1097/MD.0000000000009461

**Published:** 2017-12-29

**Authors:** Yue Jin, Jing Ying, Kai Zhang, Xiangming Fang

**Affiliations:** a Department of Anesthesiology, the First Affiliated Hospital, School of Medicine, Zhejiang University, Hangzhou; b Department of Anesthesiology, Ningbo Hospital of Zhejiang University, Ningbo, China.

**Keywords:** intubation, lateral position, upper gastrointestinal endoscopic surgery, video laryngoscope

## Abstract

**Background:**

Patients undergoing upper gastrointestinal endoscopic surgeries are generally placed in the left lateral position and require endotracheal intubation to maintain airway patency. We conducted a prospective, randomized, controlled study to evaluate the feasibility of intubation under video laryngoscopic guidance in the left lateral position during upper gastrointestinal endoscopic surgery.

**Methods:**

We compared the data of patients (n = 120) who underwent intubation under video laryngoscopic guidance in the supine or left lateral position. Patients in Group S (n = 59) were initially placed in the supine position and then shifted to the left lateral position after airway establishment. Patients in Group L (n = 61) were placed in the left decubitus position during both induction and intubation. Laryngoscopic view, intubation time, success rate, hemodynamic changes, adverse effects, and complications of intubation were compared between the groups.

**Results:**

The 2 groups showed no difference in terms of time required for intubation (Group L, 23.95 ± 4.43 seconds and Group S, 23.44 ± 4.78 seconds; *P* = .545) and number of intubation attempts. Further, the overall rate of intubation success was 100% in both groups. However, Group S exhibited significant hemodynamic changes during shift of decubitus (*P* < .001) and severe sore throat (*P* = .030). The incidences of other adverse effects such as productive cough, dryness of mouth, hoarseness, oral mucosal injury, dental injury, and hypoxia in the 2 groups were comparable.

**Conclusion:**

We concluded that intubation in the lateral position under video laryngoscopic guidance is safe and feasible performed in the left lateral position and prevents the hemodynamic change and sore throat resulting from change in decubitus.

## 1 Introduction

The rapid advances in medical technology have led to a rise in the popularity of endoscopic surgeries. In many upper gastrointestinal endoscopic surgeries, such as endoscopic mucosal resection (EMR), endoscopic submucosal dissection (ESD), and peroral endoscopic myotomy (POEM), endotracheal intubation is necessary to maintain airway patency when the patient is placed in the left lateral position. Endotracheal intubation is generally performed on the patient in the supine position, followed by a shift of decubitus from the supine position to the left lateral position. However, it is unsafe to change position of patients after the induction of anesthesia. Intubation in the left lateral can eliminate the need to change the patient's position during surgery, decrease the stress reaction and physiological interference caused by the position change of patients,^[1]^ as well as reduce the time and manpower required. Furthermore, endotracheal intubation in the lateral position may also be necessary in many emergency circumstances, including accidental intraprocedural airway loss in the lateral position, trauma, and inadequate regional anesthesia requiring conversion to general anesthesia, as well as to reduce the risk of aspiration during induction in the presence of oropharyngeal bleeding.^[2–4]^ Under these circumstances, intubation in the lateral position is important because the consequences of inadequate airway management may be catastrophic, including hypoxia, brain injury, and death.^[5]^ It can be used to immediately restore airway patency without changing the patient's position or compromising the surgical field. This is essential for the safety of patients. Therefore, anesthesiologists should be skilled in performing intubation in the left lateral position.

In standard clinical practice, endotracheal intubation is performed in the supine position to rapidly establish a patent airway. Tracheal intubation in the lateral position is difficult because the airway anatomy is distorted and the laryngeal view is compromised using the Macintosh laryngoscope.^[6]^ Further, many experienced anesthetists may be unfamiliar with performing the procedure in the lateral position.^[2]^ Moreover, in general, more attempts are required for intubation in the lateral position than in the supine position, particularly in cases of sudden intraprocedural airway loss.^[7–9]^ However, the recent improvements in video technology for intubation facilitate endotracheal intubation in the lateral position, which allows intubation without alignment of the oral, pharyngeal, and tracheal axes, as is required with Macintosh laryngoscopy. Several reports have been published on successful intubation in the lateral position using video technology, including the use of intubating laryngeal mask airway (ILMA) with the aid of a lightwand,^[10–12]^ the lightwand,^[13]^ the AirWay Scope,^[2]^ C-MAC,^[6]^ and the Flexible Fiberoptic Bronchoscope.^[14]^ Nevertheless, data on the use of video laryngoscopy guided intubation performed in the left lateral position are limited. Therefore, we conducted a randomized, prospective study to evaluate the ease, efficacy, and safety of video laryngoscopy guided intubation performed in the supine and left lateral positions.

## 2 Methods

### 2.1 Study setting

This investigation was designed as a prospective, randomized controlled study conducted across 2 tertiary hospitals (the First Affiliated Hospital of Zhejiang University and Ningbo Hospital of Zhejiang University) between June 1, 2016, and October 31, 2016 (Chinese Clinical Trial Register, ChiCTR-IIR- 15007648). The study protocol was approved by the ethics committee of the First Affiliated Hospital of Zhejiang University, Hangzhou, China. Written informed consent was obtained from all participating patients or their immediate relatives. All the intubations were performed by 2 skilled attending anesthesiologists (KZ at the First Affiliated Hospital of Zhejiang University and JY at the Ningbo Hospital of Zhejiang University). Before the trial, these 2 anesthesiologists had successfully practiced the procedure using an adult airway management trainer in the lateral position and intubated patients in the left lateral position.

### 2.2 Patients

The study population comprised all patients requiring endotracheal intubation for elective gastrointestinal endoscopic surgery in the left lateral position. The patients were allocated to the supine position group (Group S) or the lateral position group (Group L) using randomized, computer-generated numbers. The exclusion criteria were as follows: age < 18 years; American Society of Anesthesiologists (ASA) grade ≥III; known or predicted difficult airway (Mallampati score ≥3, mouth opening <3 cm, thyromental distance <6 cm); cervical spine abnormality; history of poor cardiopulmonary function (hypertension, coronary artery disease, or chronic obstructive pulmonary disease); asthma; cerebrovascular disorders; and history of surgery conducted in the pharyngeal or laryngeal region or cervical spine.

### 2.3 Procedure

**Figure 1:**
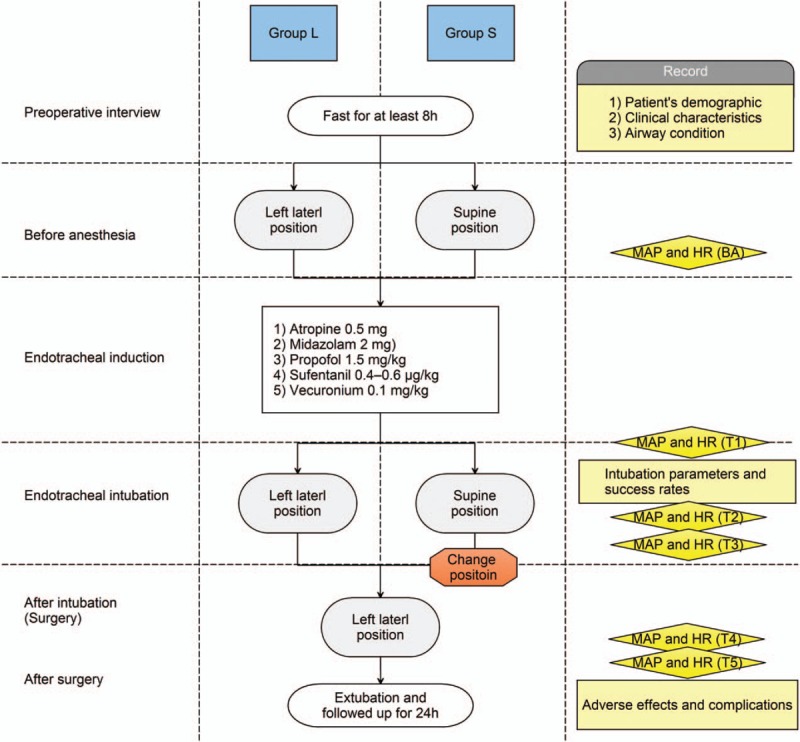
Procedure of the study and measurements applied. Group L: Endotracheal intubation with patients in the left lateral position; Group S: Endotracheal intubation with patients in the supine position.

All patients were required to fast for at least 8 hours before surgery, and no premedication was administered. On arrival in the operating room, the heart rate (HR) and noninvasive blood pressure were measured and standard electrocardiogram and pulse oximetry were monitored (Fig. 1).

**Figure 2:**
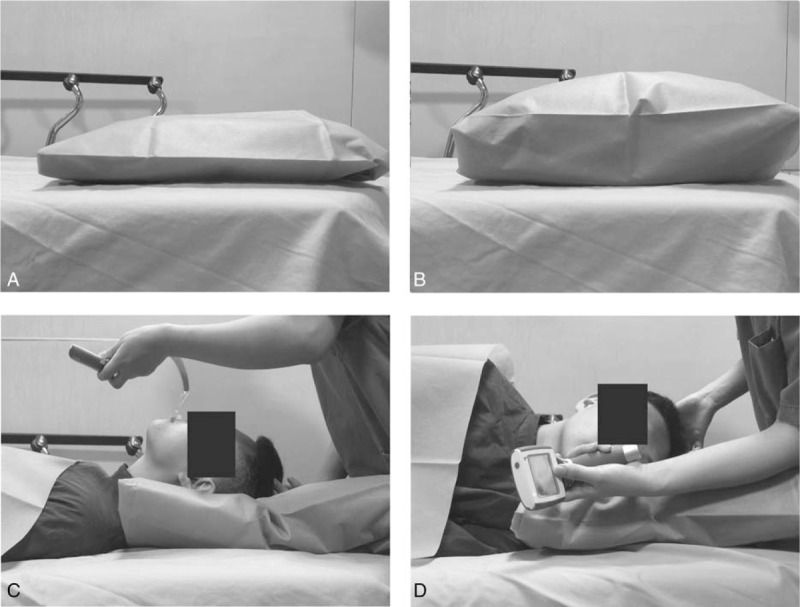
Endotracheal intubation with patients in the supine and left lateral position. A. The supporting pillow for Group S; B. The supporting pillow for Group L; C. Endotracheal intubation with patients in the supine position; D. Endotracheal intubation with patients in the left lateral position, Group L: Endotracheal intubation with patients in the left lateral position; Group S: Endotracheal intubation with patients in the supine position.

Before induction of anesthesia, the patients were positioned in the left lateral or supine position according to their group assignment. Group L patients were placed in the left lateral position during both induction and intubation. The head was placed on supporting pillows such that the sagittal axis of the head and neck was parallel to the tabletop and the neck remained extended. For patients in Group S, a single pillow was positioned under the occiput of the patient and adjusted as required to achieve a “sniffing” position until establishment of the artificial airway; then, the patients were shifted to the lateral decubitus position for the duration of the surgical procedure (Fig. 2).

For induction of anesthesia, intravenous injection of atropine (0.5 mg), midazolam (2 mg), propofol (1.5 mg/kg), sufentanil (0.4–0.6 μg/kg), and vecuronium (0.1 mg/kg) were administered. Manual ventilation was administered for 3 minutes with 100% oxygen delivered via a mask before intubation. Thereafter, an experienced anesthetist, who stood at the head of the operating table, performed intubation with a curved tracheal tube (7.0 mm internal diameter for women and 7.5 mm for men) under guidance of a video laryngoscope (TIC-SC-II; UE Medical, Taizhou, Zhejiang, China). A stable depth of anesthesia was maintained with propofol (0.1 mg/kg/min) and remifentanil (0.1 μg/kg/min). During the procedure, the end-tidal CO_2_ concentration was maintained at 35 to 40 mm Hg; the respiratory frequency, at approximately 10 to 14 times per minute; and the tidal volume, at 8 mL/kg. The airway was considered secured once a positive capnographic waveform was observed with hand ventilation in addition to visible chest movement (Fig. 1).

Any single insertion of the laryngoscope past the patient's lips was considered an intubation attempt. Intubations were attempted 3 times if necessary, with oxygenation via a face mask in the interval between the attempts. Tracheal intubation was considered a failure if not accomplished within 3 attempts. If the intubation failed, patients were shifted to the supine position and the classic laryngeal mask airway (LMA) was inserted to maintain the airway patency. At the completion of surgery and anesthesia, the ETT was removed as per the routine extubation criteria.

### 2.4 Measurement

The day before the surgery, the anesthesiologist conducted a preoperative interview and recorded the patient's demographic and clinical characteristics and airway condition, which included Mallampati score, degree of mouth opening (interincisor distance), thyromental distance (with the head extended in upright position), and sternomental distance (Fig. 1).

The following outcomes were recorded: plateau airway pressure and peak inspiratory pressure during mask ventilation; total preparation time (from arrival of patients in the operating room to the development of complications of intubation and proper position); and intubation time (defined as the time from the picking up of the video laryngoscope to confirmation of tracheal intubation by capnography. In case of multiple intubation attempts, time from the picking up of the laryngoscope for the first intubation attempt until confirmation of successful intubation by capnography was considered to be the total intubation time); required number of intubation attempts; overall intubation success rate; frequency of esophageal intubation; hemodynamic stability; and perioperative adverse effects and complications, including sore throat, productive cough, dryness of mouth, hoarseness, oral mucosal injury, dental injury, and hypoxia (defined as SpO_2_ < 95%). The Cormack–Lehane score was reported when using the laryngoscope. Intubation time was measured using a stopwatch. Hemodynamic stability was assessed by measurements of mean arterial blood pressure (MAP) and HR at the following time intervals: before induction of anesthesia (BA), after induction of anesthesia but before tracheal intubation (T1), immediately after successful intubation (T2), 3 minutes after tracheal intubation, and before the patients in Group S were shifted to the left lateral decubitus (T3), 5 minutes after tracheal intubation/after the patients in Group S were turned to the left lateral position (T4), and 10 minutes after tracheal intubation (T5). Patients were followed up for 24 hours after surgery (Fig. 1).

### 2.5 Study outcomes

The primary outcome assessed in this study was intubation time. The secondary outcomes included intubation success rate, required number of intubation attempts, hemodynamic stability, perioperative adverse effects, and complications.

### 2.6 Statistical analysis

A pilot study of 10 patients in each group was conducted to estimate sample size. The means and standard deviations (SD) of intubation time were 21.7 ± 2.06 seconds (Group L) and 24.2 ± 6.41 seconds (Group S). Thus, a minimal sample size of 58 patients per group was required with a 2-tailed α = 0.05 and a power of 80%.

Quantitative data are presented as means and SD or medians and interquartile ranges (IQR; 25th–75th percentiles), as appropriate. Qualitative data are reported as n (%). Student *t* test or Mann–Whitney *U* test was used for comparison of continuous variables such as demographics, baseline airway assessments, and intubation time. The occurrence of adverse effects and complications were analyzed using Chi-square test or Fisher exact test. Intergroup comparisons of the MAP and HR were made using repeated-measures analysis of variance (ANOVA). All analyses were conducted using SPSS 17.0 for Windows (SPSS, Inc., Chicago, IL). *P* < .05 was considered statistically significant.

## 3 Results

**Figure 3:**
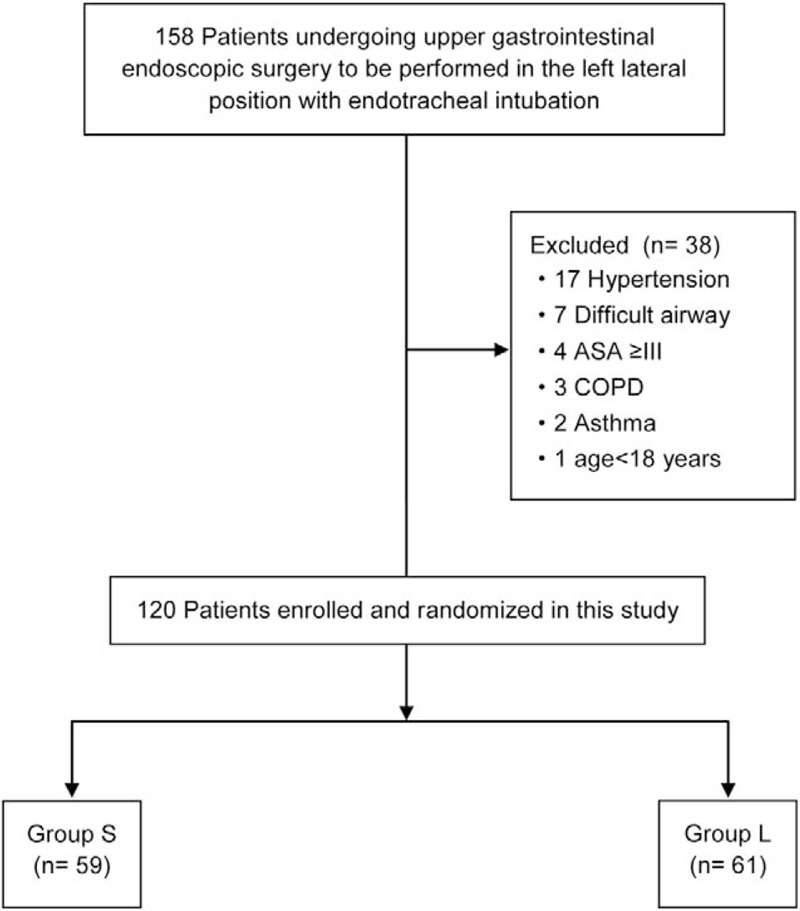
Flow diagram showing the study enrolment procedure. Group L: Endotracheal intubation with patients in the left lateral position; Group S: Endotracheal intubation with patients in the supine position. ASA = American Society of Anesthesiologists; COPD= chronic obstructive pulmonary disease.

We enrolled 158 patients who were scheduled for upper gastrointestinal endoscopic surgery to be performed in the left lateral position at either of the 2 participating centers between June 1, 2016, and October 31, 2016. Thirty-eight patients were excluded as per the abovementioned criteria. Consequently, the final sample included in the statistical analysis consisted of 120 patients—61 in Group L and 59 in Group S (Fig. 3).

Patient demographic and clinical characteristics, including age, gender, body weight, height, and body mass index (BMI), in the 2 groups were comparable. However, no significant differences were observed between the 2 groups in the results of baseline airway assessment or laryngoscopic views by video laryngoscopy, graded according to the Modified Cormack–Lehane score (*P* = .114; Table 1).

**Table 1:**
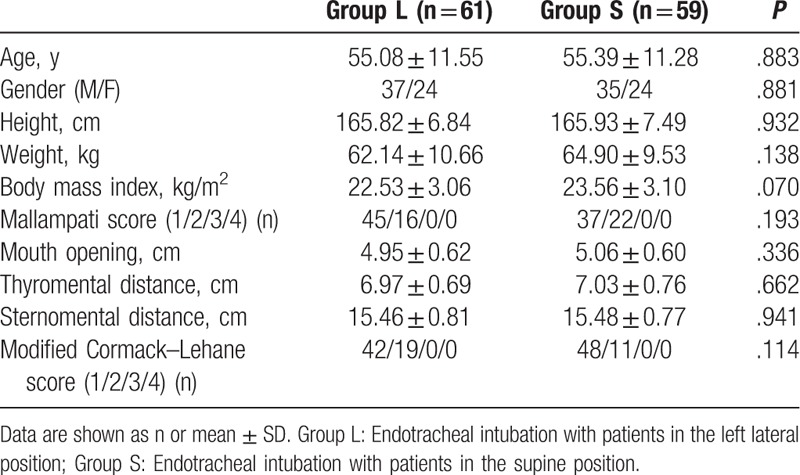
Demographic and airway assessment data.

During mask ventilation, the peak inspiratory pressure of the patients in Group L (13.10 ± 2.16 cmH_2_O) was significantly lower than that in Group S (14.59 ± 2.80 cmH_2_O; *P* = .001). Although the plateau airway pressure of the patients in Group L (9.93 ± 1.75 cmH_2_O) was lower than that in Group S (10.63 ± 2.17 cmH_2_O), the difference was not significant (*P* = .056). According to the total preparation time, patients in Group L required much less time than those in Group S (9.50 ± 1.11 vs 13.35 ± 2.42 minutes; *P* < .001; Table 2).

**Table 2:**
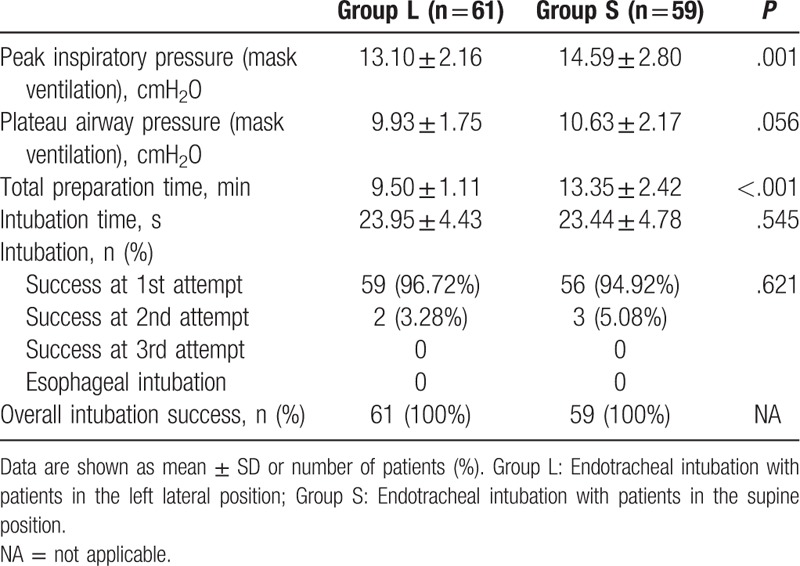
Intubation parameters and success rates in both groups.

The intubation times in Group L (23.95 ± 4.43 secondse) and Group S (23.44 ± 4.78 sconds) were comparable (*P* = .545). The success rate at the first attempt of intubation was 96.72% in Group L and 94.92% in Group S. A second attempt at intubation was required for 2 patients in Group L and 3 patients in Group S. However, the total number of intubation attempts in the 2 groups did not differ (*P* = .621). Inadvertent esophageal intubation did not occur in any of the cases. The overall rate of intubation success was 100% in both the left lateral and supine positions (Table 2).

**Figure 4:**
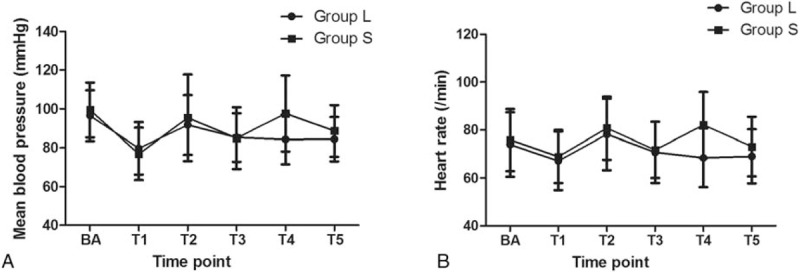
Mean arterial blood pressure (MAP) and heart rate (HR) measurements during the peri-intubation period. In both groups, the MAP values at T1 (*P* < .001), T2 (*P* < .001), T3 (P < 0.001), T4 (*P* < .001), and T5 (*P* < .001) differed significantly from the values before anesthesia (BA). Similarly, the HR values at T1 (*P* < .001), T2 (*P* < .001), T3 (*P* < .001), T4 (*P* < .001), and T5 (*P* < .001) differed significantly from those BA in both Group L and Group S., Group L: Endotracheal intubation with patients in the left lateral position; Group S: Endotracheal intubation with patients in the supine position. BA: before anesthesia induction; T1: after induction of anesthesia but before tracheal intubation; T2: immediately after successful intubation; T3: 3 min after tracheal intubation and before the patients in Group S were turned to the left lateral decubitus; T4: 5 min after tracheal intubation/after the patients in Group S were turned to the left lateral position; and T5: 10 min after tracheal intubation.

Hemodynamic changes during intubation in Group L were milder than those in Group S (*P* < .001). Compared with the levels before anesthesia, MAP decreased rapidly at T1, increased gradually by T2, and then decreased again by T3. The differences between the MAP before anesthesia and those at T1 (*P* < .001), T2 (*P* < .001), and T3 (*P* < .001) were significant, and a similar trend was observed in the case of HR (*P* < .001, *P* < .001, and *P* < .001, respectively). Between time points T3 and T4, the MAP and HR in Group S increased significantly (*P* < .001) and then decreased markedly by T5 (*P* < .001). However, the MAP and HR remained more stable in Group L (Fig. 4).

The complications of intubation observed in this study are listed in Table 3. An analysis of the adverse effects and complications showed that the supine position was associated with a severe degree of sore throat (*P* = .030). In Group L and Group S, 46 patients and 31 patients had no sore throat, 12 and 24 patients had mild pain, and 3 and 4 patients had pain on swallowing (*P* = .664), respectively. None of the patients experienced pain on both swallowing and respiration. The incidences of cough with sputum, dryness of mouth, and hoarseness were similar for both groups. None of the patients experienced complications such as dental injury or hypoxia.

**Table 3:**
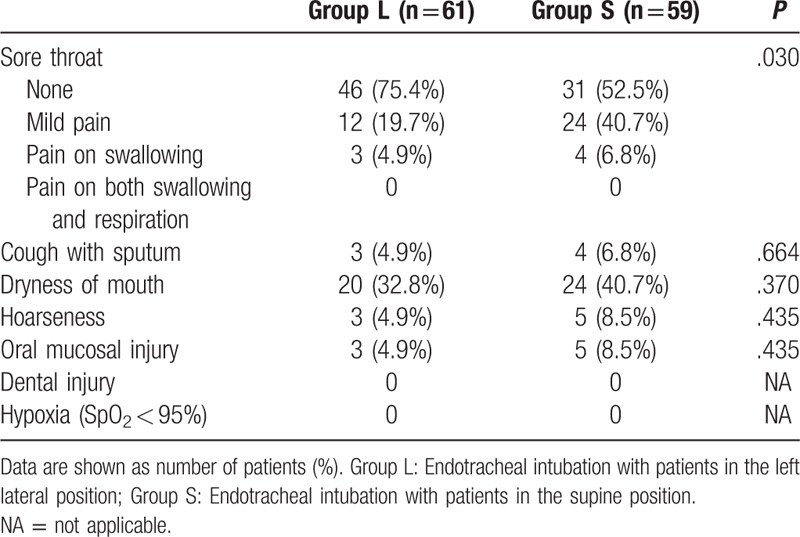
Adverse effects and complications in the 2 groups.

## 4 Discussion

In the current study, we compared the ease and safety of performing tracheal intubation under video laryngoscopic guidance in the left lateral and supine positions. Our results suggest that under laryngoscopic guidance, tracheal intubation performed by skilled anesthesiologists in the left lateral position was comparable to that performed in the supine position in terms of intubation time, number of intubation attempts, and success rate. Furthermore, the hemodynamic changes during intubation as well as the severity of sore throat were milder in Group L than in Group S. The incidences of other complications were comparable in the 2 groups. Our findings suggest that endotracheal intubation under video laryngoscopic guidance may be a safe, feasible, beneficial, and time-saving technique for patients undergoing surgery in the left lateral position.

The improvement in video technology reduces the demand for restriction in the patients’ position and facilitates endotracheal intubation of patients in the lateral position.^[2,12,13]^ In our study, we obtained comparable Modified Cormack–Lehane scores, intubation time, number of attempts, and success rate in both groups with the use of the video laryngoscope between Group L and Group S. Some previous studies have supported our results. Intubation times and success rates in the left-lateral and supine positions using Airway Scope and C-MAC video laryngoscopy were comparable. Komatsu et al^[15]^ reported intubation success rates of 100.00% versus 97.67% for the supine and left lateral positions, respectively, using an Airway Scope. Bhat et al^[6]^ demonstrated similar success rates using C-MAC video laryngoscopy (100.00% vs 100.00%). These results indicate that placement of patients in the left lateral position during surgery is safe and compatible with intubation in the left lateral position.

Our study also revealed a transient increase in hemodynamic change, which was reflected by changes in HR and MAP during the shifting of the patient's position after induction of anesthesia. This is consistent with the findings of many previous studies, which showed that a sudden change in the patient's position after induction of anesthesia may disturb their hemodynamic stability.^[1,16–18]^ However, intubation in the left lateral position was associated with a decreased hemodynamic response to change of position during general anesthesia. These changes may be well tolerated by patients with ASA Grades I and II. Notably, sudden changes in body position during anesthesia can cause accidental airway loss, airway injury, and substantial fluctuation in HR and MAP and may be life-threatening, especially in elderly, hypertensive, and obese patients.^[10,13]^ Thus, intubation in the left lateral position is necessary and highly beneficial in such cases.

In the current study, intubation in the left lateral position was associated with sore throat of less severity. Moving patients after tracheal intubation can cause airway damage. Previous studies have shown that airway complications increased during passive position change after induction of anesthesia.^[1]^ Intubation in the left lateral position may reduce the occurrence of airway adverse effects and complications due to unwarranted position change.

Induction of anesthesia in the supine position causes the tongue and soft tissue of the throat to sag downward (due to gravity), which can obstruct the airway.^[19]^ These problems are avoided in the left lateral position, which simplifies mask ventilation. As a result, the peak inspiratory pressure and plateau airway pressure of the patients during mask ventilation were lower in Group L than in Group S. Furthermore, the secretions are easier to remove from the oropharynx, which reduces the risk of aspiration during anesthesia, intubation, and extubation.^[4]^

The proficiency of the anesthesiologists may have influenced the outcome of the study. Endotracheal intubation is generally performed on a patient in the supine position. Many anesthesiologists, even those who are experienced, may be unfamiliar with the procedure for intubation in the lateral decubitus.^[2,7]^ Grosomanidis et al^[20]^ found that intubation was more difficult in the lateral position than in the supine position when using an airway management manikin. Hence, all practitioners should undergo training with a standardized demonstration via an oral presentation focusing on standard techniques required for intubation in the lateral position. It is important to note that improper positioning of the head and neck may increase the difficulty of intubation. Saini et al^[21]^ improvised a head support to facilitate endotracheal intubation in the lateral position and increase the success rate of intubation in the lateral position. Supporting the head with pillows placed on the horizontal limb to keep the head in line with the torso can prevent anatomical distortion as well as overcome the difficult in the laryngeal visibility when the patient is placed in the lateral position^[22]^ (Fig. 2). These techniques are expected to increase the success rate of intubation in the lateral position. In order to avoid any bias from this lack of familiarity, in our current study, all the intubations were performed by 2 skilled anesthesiologists who had practiced with an airway management trainer in the lateral position.

This study has several limitations. First of all, this study included a relatively small sample size. Clinical studies involving a larger population and multiple centers are required to confirm the findings of this study. Second, in order to avoid any bias from lack of proficiency, the procedures in this study were only performed by 2 skilled anesthesiologists. Both residents and attending anesthesiologists should be trained to achieve proficiency in performing intubation in the left lateral position; with the availability of more experienced staff, more large-scale studies can be undertaken to test the feasibility of the procedure. Third, patients with anticipated airway difficulties were excluded from our study. This explains why none of our patients had Grade 3 or higher scores of the Modified Cormack–Lehane scale. Thus, further studies are necessary to evaluate the feasibility of intubation in the lateral position in patients with difficult airway.

## 5 Conclusion

Our study revealed that intubation in the left lateral position may be safely and effectively performed under video laryngoscopic guidance. It can reduce the extent of hemodynamic changes and sore throat resulting from a change in decubitus after anesthesia. Therefore, we believe that anesthesiologists should acquire this skill.
